# Deference or critical engagement: how should healthcare practitioners use clinical ethics guidance?

**DOI:** 10.1007/s40592-023-00186-8

**Published:** 2024-02-29

**Authors:** Ben Davies, Joshua Parker

**Affiliations:** 1 Department of Philosophy, University of Sheffield, Victoria Street, Sheffield, S3 7QB UK; 2 Wythenshawe Hospital, Southmoor Rd. Wythenshawe, Manchester, M23 9LT UK

**Keywords:** Moral deference, Moral testimony, Ethics guidance, Professional ethics

## Abstract

Healthcare practitioners have access to a range of ethical guidance. However, the normative role of this guidance in ethical decision-making is underexplored. This paper considers two ways that healthcare practitioners could approach ethics guidance. We first outline the idea of deference to ethics guidance, showing how an attitude of deference raises three key problems: moral value; moral understanding; and moral error. Drawing on philosophical literature, we then advocate an alternative framing of ethics guidance as a form of moral testimony by colleagues and suggest that a more promising attitude to ethics guidance is to approach it in the spirit of ‘critical engagement’ rather than deference.

## Reliance on ethics guidance

At the beginning of the COVID-19 pandemic, it quickly became clear that healthcare professionals would face considerable ethical challenges. In the ethical fog, a scramble to create guidance for professionals began, aiming to support doctors facing challenging decisions in unique circumstances. As Huxtable notes, “As difficult as these questions are, professionals… are understandably looking for information and support and, fundamentally, *for answers*.” [our emphasis] (Huxtable 2020) Similarly, reports from Italy suggest that guidelines for rationing intensive care resources were produced in response to the moral distress that care workers experienced trying to carry the weight of these decisions without explicit guidance. (Rosenbaum 2020) Some authors suggested an “ethical road map” was necessary to navigate the various challenges that COVID-19 presented, and that to achieve this “there should be nationally led and coordinated development of transparent, publicly shared ethical guidance that can provide the basis for clear, consistent, and defensible decisions in all healthcare and policy settings across the country” (Fritz et al. 2020). To help fill this gap, a number of organisations produced ethics guidance, including the Royal College of Physicians (Royal College of Physicians 2020), the Royal College of General Practitioners (https://elearning.rcgp.org.uk/mod/page/view.php?id=10557), the Royal College of Psychiatrists (Royal College of Psychiatrists 2020), the British Medical Association (2020) and the United Kingdom Clinical Ethics Network (http://www.ukcen.net/covid-19/). Given the breadth of these organisations, the challenges faced by professionals go far beyond allocation decisions regarding ventilators. For instance, the Royal College of Psychiatrists considers the ethical implications of inpatients having less contact with loved ones. As important as supporting professionals is, a second rationale for producing ethics guidance was to increase the transparency and consistency of decisions, and by reducing individual bias, make decisions defensible (Huxtable 2020).

Given the ubiquity of guidance in modern healthcare, it is unsurprising that guidelines were a core part of the pandemic response. Indeed, these observations regarding the need for ethics guidance are true of many ethics guidelines (also ‘ethics protocols’; ‘professional ethics guidance’; ‘ethics guidelines’) outside COVID-19. Ethics guidance streamlines decisions, reduces decision-making burden, and can provide confidence. There may also be broader advantages. In theory, ethics guidance can be adopted across a healthcare system. Some think this makes it more likely that patients are treated equitably, reducing the role of individual clinicians’ biases (Commons and Baldwin 1997; Pattison 2001; Community Research 2017; Höglund et al. 2010), so that patients are not subject to a ‘postcode’ or ‘doctor’ lottery generated by such differences (Wilkinson and Truog 2013). Guidelines may therefore provide procedural fairness if widely adopted and robustly followed. The utility of ethics guidance makes it easy, and understandable, if doctors come to rely on it in making ethical decisions.

Our focus here is on any guidance used in healthcare to make moral decisions. Ethics guidance might be explicitly ethical, like the various algorithms and protocols designed for intensive care rationing in COVID (Savulescu et al. 2020), or implicitly ethical where values or principles are embedded within clinical guidelines. Both are included in our discussion. Ethics guidance also varies in specificity and concreteness. On the one hand, the UK’s General Medical Council (GMC) is highly specific in when and how doctors might break confidentiality (General Medical Council 2021). On the other, the approach of Beauchamp and Childress’ widely used ‘four-principles’ approach is more abstract (Beauchamp and Childress 2001).

Bioethical analysis of ethics guidance tends to focus on the guidance itself. As most clinical ethics guidance takes the form of either (1) providing a set of guiding principles, (2) outlining values that underpin a certain approach, or a mix of the two, criticism of ethics guidance is typically directed towards these aspects (Dawson 2010; Harris 2003; Hopkins 2007a; Nickel 2001a; Pattison 2001). For instance, critics may raise problems with the principles invoked or how to balance them. Alternatively, as has been the case with much COVID-19 ethics guidance, especially in terms of triage, the underlying values are critiqued. Here, we approach ethics guidance from a different perspective, setting aside the standard bioethical approaches that take issue with the *content* of ethics guidance. Rather, we make the novel step of drawing on the philosophical literature on moral testimony to focus on the attitudes that healthcare professionals should take towards guidance and how they ought to interact with it.

The examples from COVID-19 suggest one possible approach that medical professionals might take: deference. A deferential approach sees guidelines as providing *answers* about what to do. If Huxtable is correct that clinicians are looking for “answers” in ethics guidance, and they thereby follow the guidance assuming that it contains ‘the answer’, then they might assume that their actions are ethically justifiable. Providing definitive answers is one way of alleviating moral distress and reducing the emotional burdens of decision-making. The idea that guidance ensures consistency, transparency and fairness suggests that an attitude of deference is even to be encouraged. This is particularly true in cases where system-wide policies are sought since in order for guidance to guarantee the uniformity that these virtues assume, medical professionals must accept guidance as authoritative.

The idea that medical professionals might defer to ethics guidance is noteworthy because some philosophers worry about acquiring moral beliefs through testimony because of ‘the problem of moral deference’ (Boyd 2010; Crisp 2014; Fletcher 2016; Hills 2020; Hopkins 2007a; McGrath 2009; Nickel 2001a). Our paper explores the question of whether deference is a defensible approach to ethics guidance. Section 2 examines the problems with moral deference applied to healthcare ethics guidance. Section 3 sets out an alternative approach, drawing on the philosophical literature on moral testimony. This approach emphasises that ethics guidance has a place in healthcare decision-making, but that this place is, as the name suggests, to provide *advice* which can inform ethical reflection, rather than giving the ethical answer. Drawing on recent work on moral testimony (Boyd 2010; Hills 2020), we argue that healthcare professionals should approach ethics guidelines in a spirit of critical engagement, not deference.

## Moral deference: ‘just follow the guidance’

One possible attitude towards ethics guidance in professional healthcare is to see it as a ‘last word’ on ethical questions. An alternative way of viewing this attitude is that, as Huxtable puts it, that ethics guidance provides “answers” for doctors (Huxtable 2020). This form of moral deference raises several problems, exemplified by a hypothetical case:

Resuscitation: Dr Watt is caring for Mr Smith in hospital. Mr Smith was admitted with pneumonia and has a background of advanced heart failure and pulmonary fibrosis. While Mr Smith is receiving treatment for pneumonia, Dr Watt wonders whether—should Mr Smith suffer cardiac arrest—cardiopulmonary resuscitation (CPR) would be appropriate. She is familiar with the decision-making framework produced by the UK’s Resuscitation Council and uses this to determine Mr Smith’s CPR status (British Medical Association, Resuscitation Council 2016). The guideline states a DNACPR that is established on ‘clear clinical grounds’ must be communicated to the patient. Dr Watt’s view is that Mr Smith’s heart and lung condition means there is “no realistic chance that CPR could be successful”. On the basis of these clinical grounds, Dr Watt consults with Mr Smith and informs him that he is not for CPR because of the minimal chance of success and a concern that this might “prolong [his] suffering”. Mr Smith does not accept this. Dr Watt explores Mr Smith’s perspective, emphasises the risks to Mr Smith and seeks a second opinion which corroborates her own. As the guideline on CPR states that a demand for CPR does not mean the doctor is obligated to provide it, Dr Watt marks him as DNACPR. Because Dr Watt followed the guidelines at every stage, she believes she has done the right thing.

Dr Watt defers to guidance to determine what to do. She faces two moral decisions. First, should Mr Smith be marked as a candidate for CPR? This moral question may be obscured by the explicitly *clinical* nature of the guidance, exemplified by the phrases contained within the guidance like “clear clinical grounds”, “clinically inappropriate” and “a realistic chance that CPR could be successful”. Relevant clinical information alone cannot determine CPR status. To withhold CPR on “clear clinical grounds” requires evaluative consideration of what outcomes would be sufficiently beneficial to justify the intrusions of CPR. In other words, to determine whether CPR is appropriate one must identify the relevant harms and potential benefits as well as weighing them against one another. Dr Watt makes her decision to withhold CPR on the basis of the guidance, and her judgement that CPR will not be successful and may prolong suffering. Her consultation with Mr Smith may well parse some of the reasons he desires CPR, and Dr Watt may consider these in her decision, but if her view is that CPR is clinically inappropriate, the guidance supports a DNACPR. Since the need for evaluative judgement is implicit rather than explicit in the guidance, Dr Watt is enabled to implicitly rely on her own value judgements about what constitutes a successful outcome from CPR. Similarly, she does not consider what probability of success would be sufficient; she only judges that the probability is low and therefore that CPR ought to be withheld. Both types of judgement may happen in such a way that she feels she has only done ‘what the guidance says’ at each stage of the process. Dr Watt’s evaluative beliefs about outcomes and likelihood of success may strike her as obvious, natural, or incontrovertible; this increases the risk that she will not see them as morally loaded judgements at all: from her perspective, they are simply medical judgements.

The second moral decision, and second aspect of the decision-making framework in the guidance, regards the discussion that is had with the patient. The guidance here is particularly tricky. Throughout the guidance it is clear that there should be a presumption that patients are informed of a decision to withhold CPR. However, where the guidance can become confusing is in the way it distinguishes patients where CPR “will not be successful” (Sect. 5) from patients where there is a balance between the benefits and burdens of CPR (Sect. 6). It is only the latter case that invites “an open dialogue and shared decision-making between the patient and professionals” (p. 13). In the former case, where it is ostensibly obvious that CPR will not be successful, the guidance explains that there is “no legal right to treatment that is clinically inappropriate” (Sect. 5.4). In cases where the burdens *may* outweigh the benefits, the guidance seems to leave it to doctors to decide whether to assert their right to withhold clinically inappropriate interventions or to respect a desire for CPR. Using ‘clinical’ criteria, Dr Watt determines that it is clear that CPR is inappropriate, which the guidance suggests negates a need for a *discussion* with the patient, as opposed to simply *informing* them of the decision*.* The difficulty is in how to determine that burdens so clearly outweigh the benefits in the absence of a discussion with a patient. As such, obscuring the first moral question—whether CPR can be withheld on clear clinical grounds—may also obscure the second moral question about what ought to be discussed with the patient. Rather than a dialogue which provides an opportunity to explore the patient’s views on what is considered a harm or benefit of CPR and how to balance these, the only task for Dr Watt is to inform the patient.

Turning to real examples of authoritative guidance will of course raise criticisms of the *quality* of the guidance that we mentioned in Sect. 1. Indeed, what we have noted here are various issues with the framing of the guidance itself. However, we suggest that this case raises a distinctive problem of moral deference in professional healthcare, beyond mere deficiencies in the guidance. The next three subsections outline these problems in turn.

### Outcome vs. process: the moral value of decisions

One possible response to the discussion thus far is consequentialist. Peter Schaber suggests that as patients we care about our treatment and outcomes, not the inner mental life of the medical professional or the quality of their moral reasoning.^1^ If medical professionals’ deference to guidance tends towards better outcomes than does thinking for themselves, patients would surely prefer the former. Indeed, we suggested in Sect. 1, that one reason deference to ethics guidance may seem attractive is the thought that it provides clarity and consistency of decisions.

We discuss this in more detail in Sect. 3. However, we disagree that the *only* thing we care about as patients is the treatment outcome. Most patients rightfully expect that doctors take both clinical and ethical decisions seriously and trust that medical professionals will use their expertise to carefully consider their care based on their individual merits. We do not suggest that medical professionals must begin from first principles with every patient they face. Many ethical issues are reasonably straightforward for experienced clinicians, with past cases providing heuristics that may usefully guide ethical decision-making. Rather, our claim is that where there is a lack of ethical clarity, healthcare workers owe it to their patients to engage in ethical reflection.

The problem runs deeper than this, however. Even if patients expected and valued an attitude of deference from our doctors, total deference isn’t possible. Dr Watt defers to the guidance to ensure that her ‘clinical’ judgements are morally justified. Yet much of her moral decision-making occurs under the guise of ‘clinical’ indications. This has two effects. First, it simply obscures the extent to which she actually engages in moral reasoning of her own, masking it with appeal to clinical standards. So, we should care about the not only about the decisions healthcare professionals make, nor even only about their stated reasons for their decisions, but also about the processes by which they come to those decisions, because the guidance cannot do all the work for them. This leads to the second issue: when it is interpreted as providing ‘the ethical answer’, ethical guidance takes on an implicit authority that potentially offers a blanket *license* for doctors’ ethical reasoning. Dr Watt unavoidably engages in ethical reasoning of her own. But because the nature of this reasoning is not always made explicit, she comes away thinking that because she followed ‘authoritative’ guidance, whatever she did must be ethically right, or at least defensible. Rather than offering ethical guidance, then, guidance documents can risk providing tacit permission for clinicians to act on their own moral beliefs without properly engaging with those beliefs.

To be clear, we are not suggesting that following ethics guidance makes whatever decision one makes ethically acceptable. Nor are we suggesting that healthcare professionals cynically attempt to avoid responsibility under the guise of ‘following the guidance’. Rather, our concern is that some guidance obscures the extent to which medical professionals must use their own ethical judgement is required (e.g., through an appeal to purely ‘clinical’ standards), and that a purely deferential attitude to such guidance increases this risk, such that so long as the decision can be cast as following guidance, it is assumed that it must be ethically justified.

Perhaps if consulting ethics guidance always led healthcare professionals to morally justifiable decisions, taking moral problems in healthcare seriously and showing proper respect to patients would be consistent with deferring to guidance. As we suggest in the following sub-sections, though, this is not plausible.

### From ‘what should I do?’ to ‘why should I do that?’

A second problem with the level of deference that Dr Watt shows is that relying on ethics guidance risks encouraging an uncritical attitude towards ethics which, even if consistent with knowing what one ought to do in a particular instance, presents an obstacle to broader knowledge and ability. A healthcare professional who relies deferentially on guidance may do and believe they have acted in ethically justifiable way, and may be right, but still not appreciate *why* what they have done is right*.* This concern is reinforced by some of the apparent advantages of protocols listed earlier. Seeing ethics protocols as a substitute for ethical thinking, for instance, exacerbates the potential for users to lack knowledge of why one ought to act in particular ways.

One way to frame this danger is that a deferential attitude to guidance may appear to obviate the need to *critically engage* with that guidance. (Hills 2009) This would be a mistake if only for the simple reason that guidelines can get things wrong (Höglund et al. 2010; Strech and Schildmann 2011; Petrini and Farisco 2012) and deference makes one more vulnerable to replicating errors. (Nickel 2001b) This point is clear when we consider some of the inadequacies we discussed following the example of Dr Watt. We may feel that Dr Watt’s decision-making is deficient, and we may think that this reflects problems with the guidance, but we argue that it is also a deficiency in how Dr Watt *utilises* the guidance. Furthermore, we also think there would be an issue even if ethics guidelines never erred. Written guidance unavoidably operates at a general level, and those who write guidance cannot enter into dialogue with users or consider specific cases. A medical professional who understands the reasons for acting in a particular way is thus able to consider how general guidance applies to the specifics of the case before them, and indeed whether it applies at all.

Moreover, healthcare often requires the application of moral principles and values to different cases and in different ways. This is exemplified by the rapidly changing circumstances during COVID-19 and the need for clinicians to make ethically robust decisions in the absence of explicit guidance. (Fritz et al. 2020) If clinicians understand the reasons behind ethical recommendations, they are better equipped for situations not anticipated by guidance, or where guidance is lacking.

Ethical decisions in healthcare usually require not only abstract theoretical justification, but also *interpersonal* justification to patients or families, i.e., articulating justification for a decision. (Nickel 2001a; Hopkins 2007b) Medical professionals who rely uncritically on ethics guidance can explain their behaviour in the terms outlined in the guidance. But if a patient cannot understand such an explanation, or if they raise unanticipated challenges or questions, professionals who rely uncritically may struggle. If a goal of guidance is to provide transparency in decision-making, deference cannot achieve this because explaining that you followed the guidance is not enough, one must be able to articulate *why* you acted as you did. This requires critical engagement, not deference.

Moreover, even if healthcare professionals follow guidance in the ethical aspects of their decision-making, they are still responsible for the decision they make. This is one reason that it would not be sufficient to answer a patient or family with questions about a decision by pointing them to the guidance.

One might object that having theoretical knowledge about ethics raises the potential for sophistic and self-serving usage of ethical language, e.g., by applying ethical principles in a post hoc rationalisation of choices. (Schwitzgebel and Rust 2016) We acknowledge this worry. However, uncritical reliance on certain types of ethics guidance is more likely to foster this sort of error. For instance, some ethics guidance tells users to engage in ethical analysis without instruction for how this is to be done, or requires them to do some ethical thinking without explicitly acknowledging this (as in our case above). This raises considerable risk of post hoc reasoning. For instance, it is easy to see how a clinician might apply Beauchamp and Childress’s ‘four principles’ approach in such a way as to favour the clinician’s preferred course of action. (Beauchamp and Childress 2001) We do not suggest that clinicians will (necessarily) do this in a cynical way. Rather, as with our example above, the concern is that clinicians come away believing that since they have followed the ethical protocol, they must have done the right thing, ignoring the extent to which their own values and ethical beliefs influenced the outcome.

### The possibility of error

We noted above that ethics guidance can be deficient, inadequate, incomplete, and even contain errors. Even well-considered guidelines may miss issues that affect specific patients. (Strech and Schildmann 2011) For instance, early on in the COVID pandemic, the UK’s National Institute for Health and Care Excellence (NICE) issued guidance suggesting that admission to intensive care should be decided in part by reference to a ‘Clinical Frailty Scale’ (CFS). Disability rights groups quickly complained that this discriminated against patients with certain disabilities, who might be scored as highly frail due to being very dependent on others, but whose frailty was not obviously relevant to their capacity to benefit from ICU admission. In response to this criticism, NICE changed their guidance on using CFS in the COVID-19 pandemic. (Leach 2020).

An assessment of the chance of benefit from critical care is in part a moral decision. Deferring to the CFS to determine this question builds a systematic error into the decision-making process because there is a group of people whose high frailty scores result from factors that don’t track their chance of benefitting from intensive care. This is a high-stakes decision and so it is essential that doctors make the right choice. Rather than simply deferring to the NICE guidance, a doctor caring for a critically ill patient with a high score on a CFS early in the pandemic should have been prepared to think critically about the reasoning behind the use of frailty scores. Since the doctor forms a critical step in the decision-making process, they should potentially challenge the use of CFS for patients in the relevant category in order to prevent a predictable moral mistake materialising. While the guidance applies literally to such a patient, the justifying reasons for using a CFS do not.

Of course, we appreciate that it is difficult for individual medical professionals to reject national guidance even after careful reflection, especially in an emergency. And we do not mean to claim that doctors should simply do whatever they feel is right whenever they judge guidance to be mistaken. Doctors who carefully reflect on ethics guidance will sometimes face conflicts between what guidance appears to say, and what they think is right for their patient. How such conflict should be resolved may depend on factors including potential costs to patients of following guidance, and may range from outright refusal to follow guidance, to attempts to raise concerns with colleagues or those who have written the guidance. What we do suggest is that simply applying guidance with no attempt to critically reflect on its underling justification would have been ethically negligent.^2^ And of course, any ethics guidance could in principle have analogous embedded oversights.

The fact that ethics guidelines may be mistaken provides a reason for healthcare professionals to be cautious and critical in using them, considering whether the particular case before them throws up unconsidered issues, or the possibility of comprehensive error. In this way, healthcare professionals can stand in the way of errors in guidance translating into practice. Of course, since individual moral judgement can also err, it is a complex question precisely how healthcare professionals should regard particular clashes between guidance and their own considered ethical views. A clash may show the need for further consultation with colleagues, other sources of guidance, or in more difficult cases, Clinical Ethics Committees (CECs) or Clinician Ethics Advisory Groups (CEAGs), which are “multidisciplinary groups, including health professionals and lay members that…provide support for decision-making on ethical issues arising from the provision of patient care”, (UK Clinical Ethics Network 2021) and may contribute to hospital guidance and policy as well as providing advice on specific cases. We offer some expansion on this idea in the next section.

## ‘Critical engagement’ with ethics guidance

We have thus far developed a negative argument against deferential reliance on ethics guidance by healthcare professionals. We do not, however, intend this case to speak against using ethics guidance entirely. This section first develops the positive case for looking to ethics guidance, then draws further on the philosophical literature on moral testimony to develop a view of how healthcare professionals ought to engage with it.

Our positive case begins by responding to a criticism of ethics guidance. Eriksson et al. question the ‘action-guiding’ potential of guidance (2008). As we understand it, to say that a guideline is ‘action-guiding’ is to say that someone could use it to successfully work out how they ought to act. The exhortation ‘Do the right thing’ tells us how to act in some sense. But it is not even minimally action-guiding because anyone ignorant of the right course of action becomes no more enlightened by it (Lewis and Schüklenk 2021). In medicine there is often no single, clearly ‘right’ course of action given what the clinician knows; in many cases there may be several, mutually exclusive options, some of which could be reasonably justified. An instruction to ‘do the right thing’ provides no help in choosing which ‘right’ thing to do. We can contrast this with more detailed, concrete advice, such as ‘Always consult patients (or an appropriate proxy decision maker) about their treatment’. This is action-guiding to much greater degree. Although it leaves some things unclear (what exactly does ‘consulting’ patients involve?) it gives a much more concrete sense of what to do.

We focus on just one problem raised by Eriksson et al., relevant to our earlier observation that ethics guidance is limited in both specificity and responsiveness, which they call the ‘interpretation problem’. As they put it, “there will always be a gap between the rules and the practice they are meant to regulate. An agent must always interpret the rules in order to assess their applicability in a particular situation”, and for this they need some prior ethical knowledge, especially if they receive conflicting advice (2008). Eriksson et al. suggest that much guidance fails to say “anything specific about how to handle particular situations where some kind of ethical dilemma…arises”, and specifically with respect to informed consent “do not give any advice regarding how to weigh rules of consent against other ethical interests”. They conclude that “a person in need of guidance from ethical guidelines is in for a hard time”, and that guidance should be used more sparingly, to “lay down those general rules that are to be followed without exception”, but stress that in most cases “ethical competence is needed to deal with the problems as they arise”. This leaves ethics guidance as being like a lighthouse: a warning that rocky areas are to be avoided, but which provides no further help in navigating stormy seas.

As should be clear from our foregoing argument, we agree that ethical competence is a vital skill for healthcare workers. Where there is an interpretive gap there is room for bias. For instance, Dr Watt has interpreted ‘clear clinical grounds’ as ‘low probability of survival’. But even if there is a low probability, this alone does not lead to the conclusion that Mr Smith ought not to receive CPR. There needs to be an additional *moral* (as opposed to legal) argument about the disvalue of this: that it causes harm, denies the patient a ‘good death’, or is a poor use of scarce resources. And even establishing one of these moral premises leaves the additional tasks of balancing this with Mr Smith’s wishes. Nonetheless, the fact that ethics guidance does not perfectly solve the problem of bias is not in itself a reason to think that it provides no help whatsoever. Moreover, it is hard to see how leaving ethics decisions entirely up to individual doctors’ personal judgement would be an *improvement* in this respect, even if guidance by itself cannot solve the problem entirely. Simply relying on one’s own conscience magnifies the risk of motivated reasoning. (Sliwa 2012).

One might argue that there is a clear middle way here, which requires neither the use of abstract guidance, nor an epistemically unjustifiable over-reliance on one’s own conscience. This middle way is to note that medical professionals have access to *colleagues*, either in their immediate work environments (Frith 2009) or CECs.

Clinical ethics guidance has disadvantages compared with a clinician’s immediate peers or panel members (e.g., they cannot address the nuances of the case). But they also have advantages. Several authors endorse the idea of moral expertise in either a limited or general sense (Driver 2006; Jones 1999; Lillehammer 2014) and suggest that moral deference is less problematic if one is deferring to someone with genuine expertise (we do not assume that ‘moral expert’ means ‘academic’ or ‘ethicist’—as Jones notes, moral expertise about some issues is more likely to come from lived experience) (McGrath 2009; Singer 1988). While those who author them are no ethical paragons, much ethics guidance has had substantial consideration put into it by individuals with practical experience in relevant issues, who have had time for ethical reflection and engagement. As a medical professional, one’s colleagues are undoubtedly useful sources of advice; but they are also subject to similar time and other institutional pressures. CECs, on the other hand, are not available for every ethical decision that a professional may struggle with. Thus, we suggest that an appeal to peer testimony does not rule out the use of ethics guidance.

If ethics guidance is to be used by healthcare professionals, how should healthcare professionals engage with it? We suggest that one useful way to frame guidance is, rather than an authoritative source of moral truth, a form of (indirect) *advice* from colleagues in the broader medical profession. Like more immediate peers such as colleagues and panel members, those who write ethics guidance are offering a substantive opinion, itself often based on consultation with peers, about what clinicians ought to do in particular ethically difficult situations. In other words, ethics guidance is a form of moral testimony. We can explain what “moral testimony” is by considering testimony in general. While some of our knowledge is acquired directly by our senses, much of it is testimonial. The authors of this paper have true beliefs, for instance, about the date of Battle of Waterloo, the capital of Alaska, and (at least to some extent) how vaccines work. Although we have acquired all this information by testimony, almost nobody would claim that we thereby lack knowledge.

We can also acquire moral beliefs through testimony. For instance, I might come to believe that it is wrong to eat meat not by thinking about it myself, but simply by accepting the testimony of my friend who I respect. Some (though not all) philosophers have suggested that while testimony about empirical facts seems in principle unproblematic, believing things on the basis of *moral* testimony—sometimes termed ‘moral deference’—is inappropriate for competent adults (Hills 2009).

As we have argued, and as with other forms of moral testimony, reliance on ethics guidance can be deferential, where little cognitive work is performed by the user. Nonetheless, clinicians can engage with guidance in what Boyd calls a ‘co-operative’ (Boyd 2010) rather than deferential spirit. In such cases, clinicians perform considerable cognitive work by drawing connections between the content of ethics guidance and their other moral beliefs in light of the clinical context.

While we agree with Boyd, we think that even antagonistic interactions might prove useful. A doctor who was confronted by a patient who did not fit the NICE guidance early in the pandemic might have critically reflected on the reasoning behind the use of a CFS, ultimately concluding that it was wrong in the particular case before them. But to reach that conclusion responsibly, they would still have to take seriously the recommendations contained within the guidance, and think critically about the reasons behind them. Thus, we suggest that a more useful term than ‘cooperative reliance’ in this context is ‘critical engagement’. A critical attitude does not preclude cooperative reliance, but it does range more widely to include cases where one uses testimony in a constructive way despite ultimately rejecting its central claims.

In the case of healthcare ethics guidance, we take critical engagement to involve taking seriously the recommendations set out in relevant guidance and protocols, weighing them up along with other moral testimony from colleagues, and indeed from the patient and their loved ones, and using the results to inform one’s own ethical deliberation. This could involve analogical reasoning (e.g., reading a case study or hypothetical case, and seeing the connections with a current case). But it may also involve being prompted to think about a particular kind of value or consideration that had not occurred to one before. Thus, ethical guidance as testimony can play what Hills calls a ‘propagating’ role by forming part of the medical professional’s critical ethical reflection, rather than a ‘transmissive’ role in telling them what to think (Hills 2020). The precise nature of this role will depend on the form of guidance. For instance, guidance which offers a firm ethical rule, such as insistence on securing consent where a patient has capacity, could perform *both* an instructive role (telling the healthcare worker how they must behave) and, if appropriately framed, a propagating role by helping her understand why this is how she ought to behave. Our framing is likely more useful, though, in offering a distinctive way of engaging with vaguer forms of ethics guidance, such as a list of ethical considerations that might apply in a particular kind of case. Trying to view these as ‘telling me what to do’ may well lead, as we suggested above, to a masking of the ethical reasoning the user must do themselves. Framing such considerations as ethical *testimony*, on the other hand, may help to make these less determinate pieces of guidance useful. A list of items to consider (perhaps along with ways that they may be relevant) is precisely that: a set of potentially propagating ideas that may help the user reflect in different ways, and from different perspectives^3^ (Wilkinson and Dunn 2020).

### Final caveats

We have outlined an approach which views ethics guidance in healthcare as a form of ethical testimony. We end by considering two further concerns about our argument and offering some clarifications.

First, it is important to note that there are practical constraints in healthcare that limit the extent to which individuals can engage in ethical deliberation. It may be that time constraints, conflicting advice or uncertainty means that a healthcare professional struggles to make a decision. In such cases, it may be unavoidable to lean on the authority of widely accepted ethics guidance. These limited forms of deference are defensible (Jones 1999), even if it would be preferable for clinicians to develop moral understanding themselves.

A final case where moral deference may be acceptable is in cases where an individual knows that they have a poor track-record of moral decision-making, even after seeking various forms of moral advice. Tom Douglas and Peter Schaber have suggested to us that the best way to approach ethics guidance is an empirical question of what will have the best results and that in such cases the facts point against what we have recommended and in favour of moral deference.^4^ We accept in principle that such cases may form a further pragmatic exception to our general argument. However, we also note that although medical professionals should not be expected to get ethical decisions right every time, someone who truly had such poor ethical judgement might be in the wrong career just as much as someone who predictably made poor clinical decisions. Ethical reasoning is not an optional bolt-on skill for medical professionals; it is a core part of the profession. Thus, while the poor ethical reasoner may present a counter-example to our argument in the short run, in the long run they underline the importance of critical engagement and the development of moral understanding.

## Conclusion

This paper has framed ethics guidance in healthcare as a form of moral testimony. Given common philosophical worries about moral testimony, this raises the question of how healthcare professionals should relate to guidance. We examine the literature on moral deference and highlight several key issues (moral worth; understanding; error) with taking an attitude of *deference* to ethics guidelines. However, we have argued that this literature also contains the resources to develop an alternative approach through Boyd’s work on co-operative engagement, and Hills’ idea of propagation. Drawing on this work, we suggest that the appropriate attitude towards ethics guidance needs to acknowledge that healthcare professionals are often looking for answers to challenging and complex moral issues in their work, but that others may also view an attitude of deference as suspect. We propose the idea of ‘critical engagement’ as a more promising approach, though acknowledge its challenges.

A particular challenge in preparing this manuscript is the absence of empirical data on the attitude that healthcare professionals actually take towards ethics guidance. Given the wide availability of ethics guidance and the increasing volume of guidelines generally in healthcare this is surprising. Do clinicians simply defer to guidance? Do they view guidance as providing answers or is just one aspect of a bigger picture? Perhaps critical engagement describes the spirit in which clinicians rely on ethics guidance, or maybe ethics guidance is ignored as clinicians rely on their own views. A further implication of our discussion is that these questions merit empirical investigation to flesh out this important and under-investigated area of clinical ethics and practice.

## Data Availability

Not applicable.

## Footnotes

1 Conversation with authors, March 11, 2021.

2 Though the greater issue is on the part of those who developed the guidance in the first place.

3 For a related discussion of a different form of ethical advice, from CECs, see (Wilkinson and Dunn 2020).

4 Discussion with authors, March 11, 2021.
